# Emission Enhancement and Intermittency in Polycrystalline Organolead Halide Perovskite Films

**DOI:** 10.3390/molecules21081081

**Published:** 2016-08-18

**Authors:** Cheng Li, Yu Zhong, Carlos Andres Melo Luna, Thomas Unger, Konstantin Deichsel, Anna Gräser, Jürgen Köhler, Anna Köhler, Richard Hildner, Sven Huettner

**Affiliations:** 1 Organic and Hybrid Electronics, Macromolecular Chemistry I, University of Bayreuth, Universitätstr. 30, Bayreuth 95447, Germany; cheng.li@uni-bayreuth.de (C.L.); s2yuzhon@stmail.uni-bayreuth.de (Y.Z.); anna.graeser@uni-bayreuth.de (A.G.); 2 Experimental Physics IV and Bayreuth Institute of Macromolecular Research, University of Bayreuth, Universitätstr. 30, Bayreuth 95447, Germany; Carlos.Melo@uni-bayreuth.de (C.A.M.L.); konstantin.deichsel@gmx.de (K.D.); juergen.koehler@uni-bayreuth.de (J.K.); richard.hildner@uni-bayreuth.de (R.H.); 3 Centre for Bioinformatics and Photonics—CIBioFi, Calle 13 No. 100-00, Edificio 320 No. 1069 and Departamento de Fisica, Universidad del Valle, Cali 760032, Colombia; 4 Experimental Physics II, University of Bayreuth, Universitätstr. 30, Bayreuth 95447, Germany; thomas.unger@uni-bayreuth.de (T.U.); anna.koehler@uni-bayreuth.de (A.K.)

**Keywords:** perovskite solar cells, photoluminescence, intermittency, Auger recombination, ion migration, passivation, blinking, Methylammonium Lead Halide, methylammonium lead iodide

## Abstract

Inorganic-organic halide organometal perovskites have demonstrated very promising performance for opto-electronic applications, such as solar cells, light-emitting diodes, lasers, single-photon sources, etc. However, the little knowledge on the underlying photophysics, especially on a microscopic scale, hampers the further improvement of devices based on this material. In this communication, correlated conventional photoluminescence (PL) characterization and wide-field PL imaging as a function of time are employed to investigate the spatially- and temporally-resolved PL in CH_3_NH_3_PbI_3−x_Cl_x_ perovskite films. Along with a continuous increase of the PL intensity during light soaking, we also observe PL blinking or PL intermittency behavior in individual grains of these films. Combined with significant suppression of PL blinking in perovskite films coated with a phenyl-C61-butyric acid methyl ester (PCBM) layer, it suggests that this PL intermittency is attributed to Auger recombination induced by photoionized defects/traps or mobile ions within grains. These defects/traps are detrimental for light conversion and can be effectively passivated by the PCBM layer. This finding paves the way to provide a guideline on the further improvement of perovskite opto-electronic devices.

## 1. Introduction

Together with the unprecedented development of solution-processed inorganic-organic halide organometal perovskite-based solar cells (e.g., CH_3_NH_3_PbI_3−x_Cl_x_ and CH_3_NH_3_PbI_3_), with power conversion efficiency (PCE) evolving from 3.8% [1] to 20.1% [2], the characterization of these materials has also made significant breakthroughs in the last few years [3]. A number of different methods are employed, ranging from crystallographic study [4,5], photo-physical investigation [6,7,8], to electrical characterization [9,10], etc. Presently, both scientific and technical interests concentrate on how to further improve PCE and decrease the energy loss during the light conversion process. However, till now, there is still lack of a general guideline and evaluation to characterize the quality of perovskite films, especially at a microscopic scale.

Among various characterization approaches, photoluminescence (PL) measurements [6,11], in particular wide-field PL imaging [12,13,14,15], have been demonstrated to be powerful tools to reveal the underlying physics in perovskite materials, such as the distribution of defects, charge carrier lifetimes, analyzing recombination processes, etc. In general, PL characteristics of perovskite films are closely connected with its quality, in terms of the charge carrier lifetime and the recombination pathway [16]. In detail, a slow PL decay, or long carrier lifetime, is associated with less unintentional doping or defect states inside a domain, which lead to unfavorable non-radiative carrier recombination pathways [14]. Charge carrier recombination is considered as a combination of (1) trap-/defect-assisted (Shockley–Read–Hall recombination, via the sub-bandgap traps) (first order); (2) free electron-hole bimolecular (second order) and (3) Auger recombination (third order) [17,18]. Among them, radiative bimolecular recombination would be preferable as it facilitates approaching the maximum PCE, i.e., the Shockley–Queisser limit [19].

Although individual perovskite nanoparticles have been intensively studied by confocal PL microscopy [13,20,21], the knowledge on the perovskite film (i.e., ensemble of perovskite grains) [15] is still far from being fully understood. This is mainly due to the complex boundary conditions, broad distribution of particle sizes and trap sites [14]. In this communication, therefore, we apply both conventional PL characterization and the spatially-/temporally-resolved PL imaging to investigate perovskite films, revealing the possible factors hindering PCE towards the Shockley–Queisser limit.

## 2. Result and Discussion

**Figure 1:**
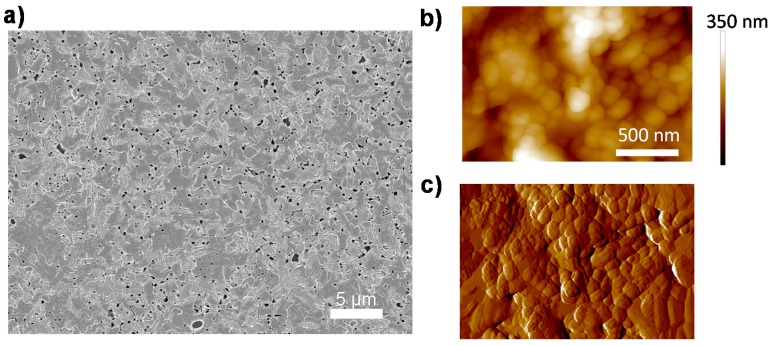
Morphology characteristics of a CH_3_NH_3_PbI_3−x_Cl_x_ perovskite film. (**a**) Scanning electron microscopy (SEM) image; (**b**) atomic force microscopy (AFM) morphology image; and (**c**) AFM phase image. The color bar in (**b**) indicates height.

The CH_3_NH_3_PbI_3−x_Cl_x_ perovskite films, which are investigated in this communication, are prepared by spin-coating of a mixed halide precursor solution (CH_3_NH_3_I:PbCl_2_ = (3:1)) on quartz glass substrates, followed by a thermal annealing step in a nitrogen glovebox. The detailed fabrication process has been described in previous work [22]. The morphology of the film is shown in Figure 1. A uniform perovskite film is deposited on quartz substrate with only minor pinhole density due to gas release during annealing [23] (Figure 1a). We also employ atomic force microscopy (AFM) to investigate the microscopic structure of the film, shown in Figure 1b,c. It is evident that the perovskite film is comprised of densely-packed grains, which are ranging from 100 nm to 800 nm in size.

**Figure 2:**
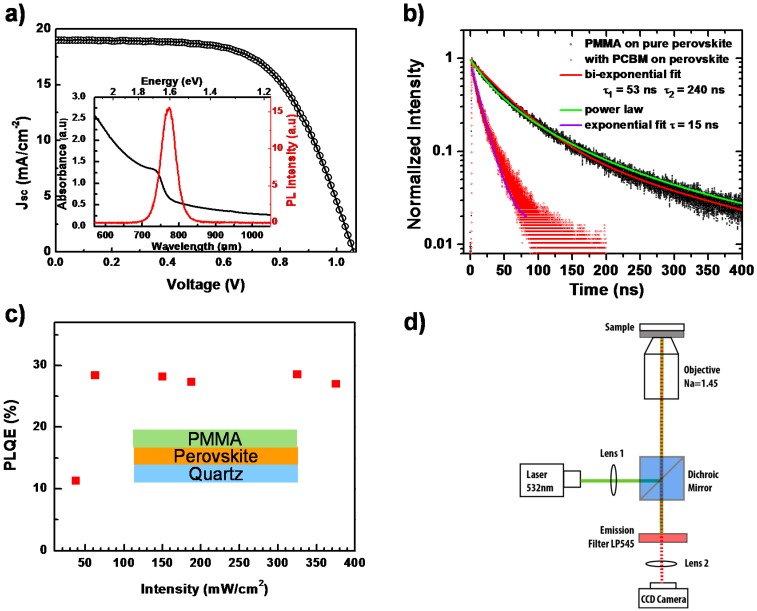
(**a**) Light current-voltage (*J–V*) curve measurement of a perovskite solar cell. The inset shows the photoluminescence (PL) and UV-VIS absorption spectra of a perovskite film; (**b**) Time-resolved photoluminescence measurement on a perovskite film with (red dots) and without (black dots) a PCBM quencher layer, together with fit lines; (**c**) Photoluminescence quantum efficiency (PLQE) of a perovskite thin film as a function of laser intensity. The inset shows the schematic of the device; (**d**) Schematic diagram of the PL imaging microscope.

The UV-VIS optical absorption and PL spectra of CH_3_NH_3_PbI_3−x_Cl_x_ perovskite thin film, measured with commercial spectrophotometers at room temperature are shown in the inset of Figure 2a. The absorption edge is located at around 769 nm, which is consistent with previous other papers [24]. For the PL properties, being excited by a 532-nm laser, the emission peak is centered at around 780 nm. This small Stokes-shift (energy difference between optical absorption and emission), unlike the one in organic materials, is attributed to the small vibrational relaxation in perovskite [6]. Incorporating this perovskite film into a typical perovskite solar cell architecture (Figure S1) results in a performance as shown in Figure 2a. From the light current-voltage (*J–V*) curve measurement, we obtain the open circuit voltage *V*_oc_ = 1.06 V, short circuit current *J*_sc_ = 18.9 mA/cm^2^ and fill factor FF = 62.3%, and, hence, the PCE is calculated as 12.5%.

By monitoring the PL decay after photoexcitation, we can investigate the charge carrier recombination kinetics. Figure 2b presents the normalized time-resolved PL behavior of this perovskite film under pulsed laser excitation at 485 nm, with and without a phenyl-C61-butyric acid methyl ester (PCBM) layer, respectively. The PL decay of the pure perovskite film cannot be described by a mono-exponential decay. Rather, it can be described using a power-law dependence. The best bi-exponential fit would give a dominant short decay component of about 52 ns.

Solid lines in Figure 2b represent the fits to bi-exponential decays, power-law decays and to exponential decay. For the quenched PL decay with PCBM, a diffusion model as described by Stranks et al. [6] should actually be applied. If one uses, for simplicity, an exponential fit, a decay time of 15 ns is obtained. We also carry out steady-state photoluminescence quantum efficiency (PLQE) measurements [25] on perovskite films with a PMMA layer as a function of the excitation intensity using a wavelength of 485 nm to study the recombination. As shown in Figure 2c, in the initial low excitation intensity regime, PLQE rises with the laser intensity. We consider that this increase is associated with the possible trap-filling process, as well as the increase of exciton density as the photogenerated species [17,26]. In detail, due to the low temperature solution processing of the perovskite film, there can be various defect states within the crystallized bulk and surface, such as vacancies, interstitials, substitutions, etc. [27,28,29]. These defects can act as trap-assisted recombination (Shockley–Read–Hall recombination) centers, via which the free charge carriers can undergo non-radiative recombination processes [30]. These recombination centers would lead to shorter carrier life times and a lower open circuit voltage (*V*_oc_). When illuminated, these sub-bandgap traps would be filled and stabilized by photogenerated electrons/holes, reducing the non-radiative recombination probability and consequently increasing *V*_oc_ under light soaking [31,32]. For the higher excitation intensity, the PLQE would reach a saturated value, indicating that all traps associated with the non-radiative recombination have been filled [17,26,33]. This scenario is consistent with our experimental data. However, we note that the PLQE is still quite low, around 30%, which is supposed to approach unity according to the detailed balance model [19,34]. This implies that besides the trap-assisted recombination, there exists an additional non-radiative recombination pathway. To understand the detailed mechanism, which suppresses the further increase of PL intensity, we employ spatially- and temporally-resolved PL microscopy to investigate the perovskite film locally on the level of individual grains.

The detailed experimental setup, which is displayed in Figure 2d, has been described in a previous paper [35]. Briefly, we employed a home-built microscope, which can be operated using wide-field illumination, and a charge-coupled device (CCD) camera as detector to image the PL of large areas (diameter ~60 µm) of a sample. We measured typically sequences of up to 2000 PL images with exposure times as short as 50 ms per image, which allows us to follow temporal changes of the PL intensity from the perovskite film under continuous laser illumination at a wavelength of 532 nm. Here, to rule out the possible influence of environmental effects on the perovskite films, i.e., oxygen and water molecules [36,37], we spin-coated a polymethyl methacrylate (PMMA) layer with a ~200-nm thickness on the top of the perovskite film as the protection layer. In addition, the PMMA layer was in direct contact with the immersion oil of the microscope objective during the whole PL characterization, which further prevents oxygen from diffusing into the film.

**Figure 3:**
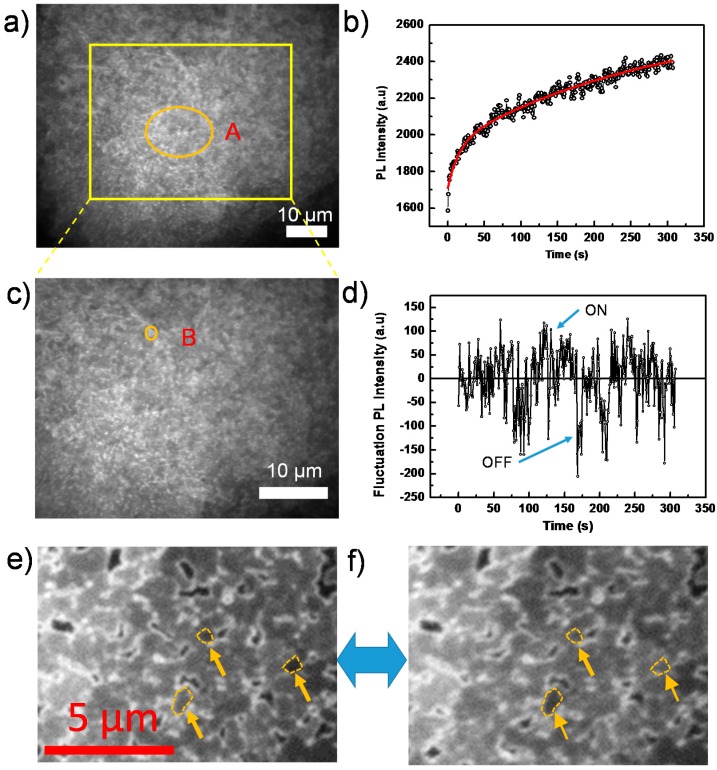
(**a**) Wide-field PL image of a perovskite film taken from a sequence of 400 consecutively-recorded images with an exposure time of 50 ms, an interval time of 500 ms and an excitation intensity of 44 mW/cm^2^; (**b**) PL intensity trajectory extracted from Area A in the sequence of images in (**a**). The red line is the fit by an exponential function; (**c**) Enlarged view of the yellow square area of (**a**); (**d**) PL intensity trajectory extracted from Area B in (**c**) after subtraction of the exponentially increasing base line; (**e**,**f**) Individual grains in “ON” and “OFF” states, respectively, indicated by yellow arrows.

Figure 3a is an example of a wide-field PL image out of a sequence of images from a perovskite film, which agrees generally with the SEM result, showing ensembles of grains on the film and the appearance of pinholes on the surface. Note that, due to the diffraction limit, grains with a size smaller than ~300 nm cannot be resolved with our microscope and, thus, appear as blurred structures. However, we still observe the existence of dark crystal grain/particle boundaries. The observation of dark grain boundaries (non-radiative recombination centers) has also been observed by higher resolution confocal microscopy, as demonstrated by deQuilettes et al. [14] for example.

Figure 3b shows the PL intensity as a function of time, obtained by extracting the integrated PL of the orange circled Area A in Figure 3a from each image of the sequence. We find that the PL intensity continuously increases during the light soaking process, as shown in Figure 3b. This increase, which agrees with the previous PLQE measurement, has also been reported in other papers [17,21,38] and is attributed to trap filling processes.

Here, we note that the time dependence of the PL intensity in Area A, shown in Figure 3b, is fitted well by a bi-exponential function with time constants of ~14 s and ~280 s and prefactors of 230 and 690, respectively. Higher excitation intensities render shorter time constants, which is shown in more detail in the Supplementary Materials (Figure S2). This implies that there exist two distinct trap-filling processes, that is a quick one and a much slower one. We propose that the quick process is associated with the direct filling of defect states in the perovskite film [20]. These defects originate from the symmetry breaking of the perfect bulk crystalline structure in the vicinity of the surface or grain boundary, where well-defined facets are lacking [29]; while, for the slower one, we assume that it is ascribed to the formation and migration of defect states in the perovskite film under light illumination. Recently, more and more studies have been carried out in this field, investigating the roles of defect formation/migration on the hysteresis and long-term (seconds to minutes) phenomena [39,40,41]. Hoke et al. [42] observed the presence of an iodine-rich phase in mixed halide perovskite under light irradiation. Chen et al. [43] detected the light activation and accumulation of ions by light soaking, resulting in PL quenching in the perovskite film. Yuan et al. [44] attributed the degradation of perovskite structures to the ion migration via light or external electron beam. Hentz et al. [45] also observed the formation of an iodine-rich region induced by an external electron beam.

The overall continuous PL intensity enhancement, as shown in Figure 3b, is superimposed by strong PL intensity fluctuations beyond experimental noise. This behavior is reminiscent of random switching between ON (highly emissive state) and OFF (weakly emissive state) in the emission trajectory, which is known as blinking or PL intermittency [46]. The intermittency behavior is demonstrated in the Supplementary Materials (Figure S3), which shows the long-term OFF state. In addition, the video in the Supplementary Materials also clearly indicates the blinking behavior confined within individual grains. Although the blinking behavior has been observed in perovskite nanocrystals [13,21,47], it is still not fully studied on compact films composed of the densely-packed perovskite grains [15].

To reveal the underling mechanism, we investigate individual grains, thus avoiding averaging over ensembles of grains in the film. Figure 3c displays an enlarged view of the yellow boxed area in Figure 3a, and an individual grain is highlighted by the orange circle labeled with B. As shown in Figure 3d, the PL intensity trajectory of this grain B, after subtraction of a continuous bi-exponentially-increasing baseline, shows a typical blinking behavior (see Supplementary Materials, Figure S3 for the individual grain in a shorter time scale), that is random distribution of ON/OFF states in the PL intensity trajectory. More individual grains exhibiting “ON” and “OFF” states are shown in Figure 3e,f, and the Supplementary Materials (Figure S3). Note that some ON or OFF states even last for more than 20 s, which indicates a significantly slow dynamic process.

**Figure 4:**
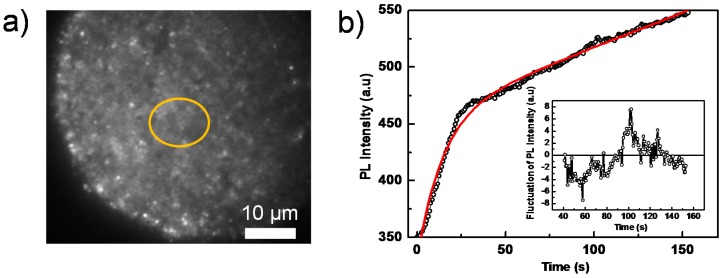
(**a**) Wide-field PL image of a perovskite film, covered with a PCBM quencher layer, taken from a sequence of 200 consecutively-recorded images with an exposure time of 100 ms, an interval time of 500 ms and an excitation intensity of 280 mW/cm^2^; (**b**) PL intensity trajectory extracted from the yellow circled area in the sequence of images in (**a**). The red line is the fitting line by a bi-exponential function. The inset shows the relative fluctuations of the PL intensity after subtraction of the bi-exponential fit function.

To further investigate the detailed processes giving rise to blinking, we performed PL imaging on perovskite films coated with a PCBM layer, which acts as a PL quencher layer. Compared to the pure perovskite film, the overall PL intensity reduces significantly despite using higher excitation intensities (Figure 4a, which shows a PL image out of a sequence of images). This PCBM layer effectively separates the photogenerated charge carriers (free electron/hole or weakly bound excitons) [48], because PCBM serves as an electron acceptor and consequently quenches the radiative charge recombination (Figure S4). Figure 4b shows the integrated PL intensity of the yellow circled area in Figure 4a as a function of time, which can be well fitted by a bi-exponential function with time constants of 5.4 s and 14.6 s, respectively. This is consistent with the results displayed in Figure 2b, in which a faster (quenched) PL decay is due to the PCBM quencher layer. It is interesting to note that although the overall PL still increases as a function of time, the blinking amplitude significantly reduces below the noise level (the ratio between the fluctuation and the average emission intensity), both in the whole film and in individual grains, as shown in the inset of Figure 4b.

**Figure 5:**
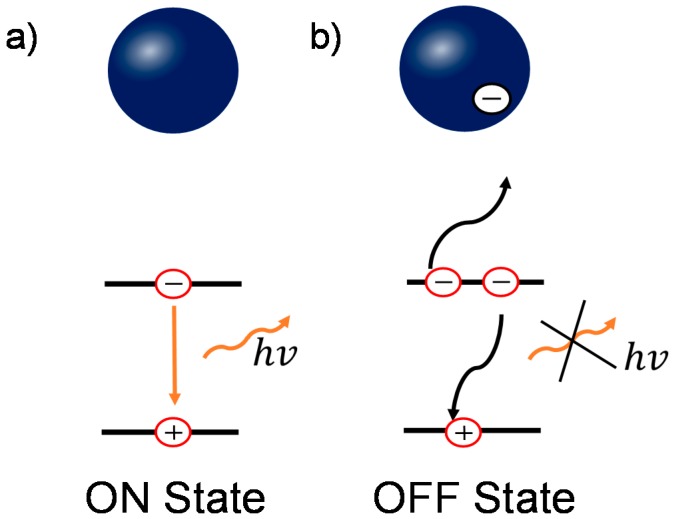
Schematic diagrams of the relationship between the charged grains and blinking behavior in perovskite. (**a**) In the uncharged states, the dominant recombination pathway is bi-molecular recombination; (**b**) In the charged states, the non-radiative three-carrier Auger recombination plays an important role. Blue ball represents an individual perovskite grain.

Based on the previous results, combining both the conventional PL characterization and wide field PL imaging, we can reveal the underlying physics in the enhancement and quenching of the PL. Though there are several models to interpret the blinking behavior [46,49,50], the presence of OFF states in perovskite film/nanoparticles is commonly attributed to additional charges due to charge trapping process [15,20,21]. When there is no charge trapped in perovskite grains, shown in Figure 5a, the dominant decay process is bi-molecular recombination (for medium carrier densities) [17], which is radiative during the recombination process, denoted as the “ON” state. In contrast, when photogenerated charges are trapped in the grain, either by surface or bulk defect states, the ionized surrounding enhances Auger recombination [15,51]. This recombination involves a recombination of an electron and a hole, followed by a process of energy transfer to a third carrier instead of photon emission, as shown in Figure 5b. This process is non-radiative and therefore renders the grains dark in PL imaging, denoted as the “OFF” state. When these trapped charges release, the PL emission recovers to the “ON” state. This Auger recombination statistically reduces the PL intensity in the whole film, resulting in the loss in *V*_oc_ and PCE [52,53].

Note that the long durations of more than 20 s of “ON” and “OFF” states are similar to the slow response in electrical transient behavior, such as hysteresis and light-induced degradation [15,44]. This implies that it can be associated with the same mechanism, i.e., ion migration. These ions can be driven by the external electrical field and consequently accumulate, enhancing the Auger recombination locally [15]. In addition, these ions are also able to migrate between grains within the film [15,41].

When the perovskite film is covered with a PCBM quencher layer, the PL blinking is significantly suppressed (insets in Figure 4b). This is ascribed to two possible reasons: First, PCBM has been demonstrated to be a good candidate to passivate traps in perovskites, leading to a charge de-trapping process [54]. In addition, the insertion of PCBM suppresses the ionic migration among the grains of perovskite, leading to a further reduction of PL blinking [55]. Second, the Auger recombination is proportional to the third power of charge carrier density *n*^3^ [16]. Owing to the effective charge transfer process at the perovskite/PCBM interface, the negative charge carrier density (*n_e_*) inside the perovskite significantly decreases, giving rise to the decrease of the Auger recombination contribution.

## 3. Experimental Methods

CH_3_NH_3_I (MAI) was purchased from Tokyo Chemical Industry (TCI Deutschland GmbH, Eschborn, Germany), and all other chemicals were purchased from Sigma-Aldrich and used as received.

### 3.1. Perovskite Film Fabrication for PL Experiment

Glass substrates were washed and cleaned with acetone and isopropanol for 10 min each in ultrasonic baths. Then, these glass substrates were treated within an ozone chamber for approximately 10 min. Following that, in a nitrogen glovebox (both water and oxygen less than 10 ppm), the perovskite precursor, i.e., MAI and PbCl_2_ (3:1) in anhydrous *N*,*N*-dimethylformamide (DMF), was spin-coated on glass substrates at 3000 rpm for 60 s. Then, these as-spun films were annealed at 100 °C in the nitrogen atmosphere for 60 min. Subsequently, 20 mg/mL phenyl-C61-butyric acid methyl ester (PCBM) dissolved in chlorobenzene were coated on the film at 2000 rpm for 30 s as a quencher layer. In the end, 40 mg/mL poly(methyl methacrylate) (PMMA) dissolved in butyl acetate (anhydrous, 99%) was spin-coated on the perovskite film at 2000 rpm for 60 s acting as a protection layer.

### 3.2. Conventional PL Characterization

#### 3.2.1. PLQE Measurements

Photoluminescence quantum yield (PLQE) was taken using an integrating sphere and a laser diode at a 485-nm wavelength (PicoQuant GmbH, Berlin, Germany). The spectra are spectrally corrected for grating, charge-coupled device (CCD) and fiber efficiencies.

#### 3.2.2. Time-Correlated Single Photon Counting

PL transients are measured with a time-correlated single photon counting (TCSPC) setup (FluoTime 200, PicoQuant GmbH). The excitation source was a pulsed laser diode with a 485-nm wavelength with 2- to 10-MHz repetition rate and a pulse duration of about 140 ps.

### 3.3. PL Imaging Setup

The setup used for PL imaging of perovskite films is based on a home-built confocal microscope. The excitation source was a pulsed diode laser (LDH-P-FA-530L, 20-MHz repetition rate, 70-ps pulse duration, PicoQuant GmbH). This laser beam was spatially filtered and directed to the microscope equipped with an infinity-corrected high-numerical-aperture oil-immersion objective (Plan Apo, 60×, numerical aperture 1.45; Olympus, Japan). The perovskite film was placed in the focal plane of the objective, and the sample position was controlled by a piezo-stage (Tritor 102 SG, piezosystem Jena GmbH, Jena, Germany). In order to homogeneously illuminate a large area (diameter ~60 µm) of the films, we additionally inserted a wide-field lens in the excitation beam path that focuses the laser light into the back-focal plane of the objective.

The PL signal was collected by the same objective, passed a long-pass filter (LP545, AHF analysentechnik AG, Tübingen, Germany) to suppress residual laser light and was imaged onto a CCD camera (Orca-ER, Hamamatsu, Japan) by an objective lens. We have done an estimation on the noise level of the PL intensity measurement as follows. The laser power stability is better than 3% (r.m.s.); to be conservative, we use 3% in the following, i.e., *s_Laser_* = 0.03. The CCD’s dark current amounts to 0.03 electrons per pixel per second, i.e., with the used exposure times of <100 ms, this dark current is <0.003 electron per pixel per exposure. As will be clear from the numbers calculated below, this dark current is negligible and will not be considered further. The readout noise of the CCD-camera is 8 electrons (r.m.s.). The measured and displayed signals are given in AD counts (i.e., after signal amplification and AD conversion). The resulting conversion factor is 4.6 electrons per AD count according to the manufacturer. Finally, the created electrons have to be converted into detected photons to estimate the photon shot noise contributing to the signal. In the emission range of the samples around 800 nm, the quantum efficiency of the CCD amounts to 30%. For the data shown in Figure 3b,d, we estimate the noise level in the high signal regime starting at around 100 s. Here, the average signal is 2200 AD counts, corresponding to 10,120 electrons or 33,700 photons. For the readout noise, we then obtain *s_CCD_* = 0.0008. From the number of detected photons, the shot noise is *s_Shot_* = 0.005. Hence, in total, the noise level is $s\;=\;\sqrt{s_{Laser}^{2}+\;s_{CCD}^{2}\;+\;s_{Shot}^{2}\;}\;=\;0.03$, which translates into 67 AD counts. Comparing this noise level to the observed signal fluctuations of about ±150 AD counts in Figure 3d, it is clear that these fluctuations cannot arise from noise. For the data shown in Figure 4, the situation is slightly different: using the displayed AD counts of around 500 in the high signal regime starting around 80 s, we obtain a noise level of 16 AD counts following the same procedure as above. Hence, in the presence of the PCBM quencher layer, the observed fluctuations are largely determined by the noise level in these experiments.

## 4. Conclusions

In summary, by employing the conventional PL and wide-field PL image characterization, we have observed the enhancement and fluorescence intermittency (PL blinking) in a mixed halide perovskite film. We attribute the PL enhancement to the trap-filling process. In the meantime, we suggest the PL blinking behavior to the enhanced Auger non-radiative recombination due to the additional charges within the grain. These charges possibly originate from the photogenerated charge carriers trapped by defects or the mobile ionized defects (e.g., iodide ions or iodide vacancies). This photoionized process results in the PL blinking, hindering the approach towards the Shockley–Queisser limit. Therefore, this finding provides unique insight to a guideline on how to further improve the PCE of perovskite solar cells.

## Supplementary Materials

Supplementary materials can be accessed at: http://www.mdpi.com/1420-3049/21/8/1081/s1.
