# Supplementary material for: Emission Enhancement and Intermittency in Polycrystalline Organolead Halide Perovskite Films
Source: Molecules. 2016 Aug 18;21(8):1081. doi: 10.3390/molecules21081081 (PMC6274434; doi:10.3390/molecules21081081)
Supplement: Supplementary file 1 [file molecules-21-01081-s001.zip › molecules-138581-supplementary/molecules-138581-supplementary-english corrected no markup.pdf]

Supplementary Materials: Emission Enhancement and Intermittency in Polycrystalline Organolead Halide Perovskite Films

Cheng Li, Yu Zhong, Carlos Andres Melo Luna, Thomas Unger, Konstantin Deichsel, Anna Gräser, Jürgen Köhler, Anna Köhler, Richard Hildner and Sven Huettner

1. Device Structure

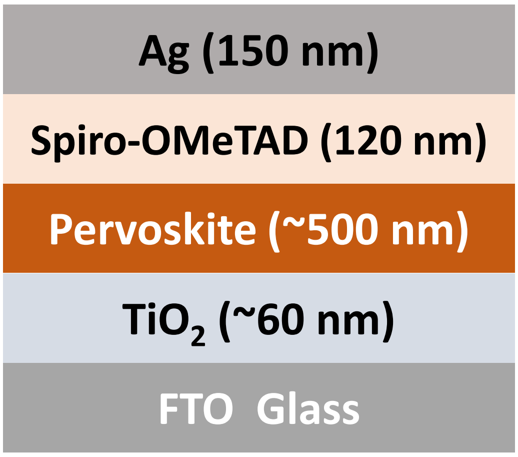

Figure S1. The schematic diagram of a typical perovskite solar cell.

2. Evolution of Photoluminescence at Different Excitation Intensities

A pure perovskite film on glass (like in Figure 3) was measured under low (44 mW/cm², Figure S2a,c) and higher excitation intensity (280 mW/cm²; Figure S2b,d). Both curves were fitted by a bi-exponential function, showing a much faster increase process with time constants of 4 s and 120 s at the higher excitation intensity.

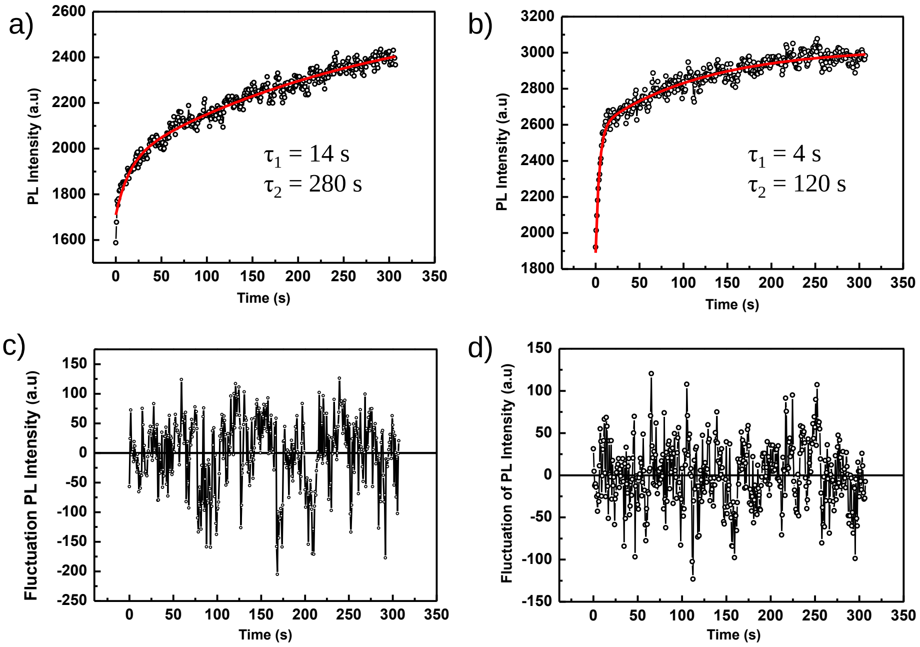

Figure S2. Comparison of PL intensity trajectory extracted from the same device in the sequence of images with excitation intensities of (**a**) 44 mW/cm² and (**b**) 280 mW/cm², respectively. The red lines are the corresponding bi-exponential fits; (**c**,**d**) are the fluctuation of the PL intensity under excitation intensities of 44 mW/cm² and 280 mW/cm², respectively.

3. Blinking in Detail

A video file is available for download, showing the PL intermittency in the pristine $CH_3NH_3PbI_{3-x}Cl_x$ perovskite film (excitation intensity 44 mW/cm²). The sequence is played 1.4x faster than real time. The diameter of the illumination spot is ~60μm.

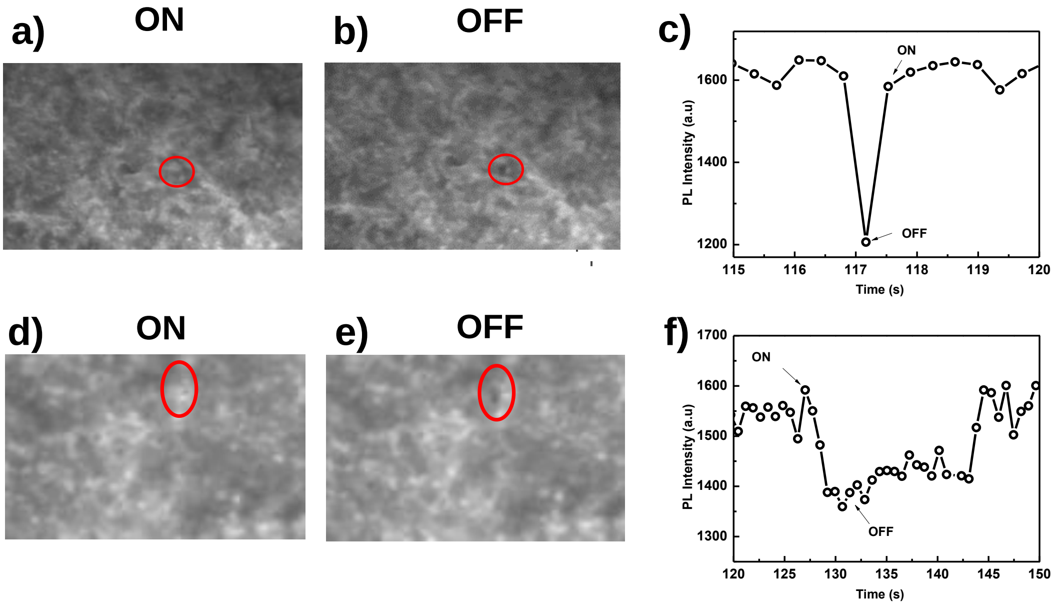

Figure S3. Different grains with PL blinking behaviors. (**a**) to (**c**) are one single grain with short OFF states; (**d**) to (**f**) are one single grain with longer OFF states.

4. Photoluminescence Quantum Efficiency

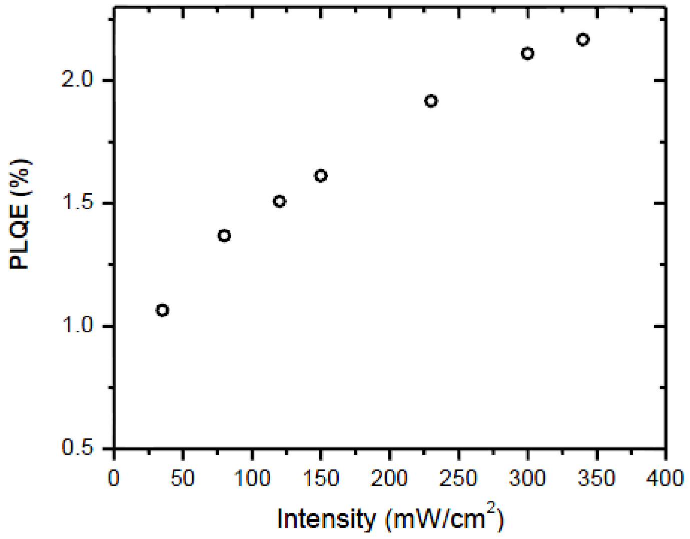

Figure S4. PLQE measurement of the perovskite/PCBM device, which exhibits PL quenching compared to the one without the PCBM quencher layer.
